# Exploring Minimum Secondary Injury for the Treatment of Ocular Trauma With Giant Intraocular Foreign Bodies

**DOI:** 10.3389/fmed.2021.800685

**Published:** 2022-01-28

**Authors:** Jing Ma, Xiaofang Zhang, Xuemin Jin, Wenzhan Wang

**Affiliations:** Department of Ophthalmology, Henan Provincial Ophthalmic Hospital, Henan Ocular Trauma Institute, The First Affiliated Hospital of Zhengzhou University, Zhengzhou, China

**Keywords:** intraocular foreign body, giant, secondary injury, extraction, surgery, ocular trauma

## Abstract

**Purpose:**

To investigate the clinical therapy for giant intraocular foreign bodies (IOFBs) and evaluate the best treatment method with minimum secondary injury.

**Methods:**

We retrospectively analyzed the data of 73 eyes of 73 patients with ocular trauma caused by giant IOFBs between January 2016 and December 2018. The IOFB size, localization, shape, and magnetic properties were recorded. The best corrected visual acuity (BCVA), ocular tissue injuries, entrance wound, interval time from injury to second phase surgery, silicone oil removal, and globe recovery were also observed. The cases were divided into three groups based on the following IOFB extraction paths: limbus path, the pars plana path, and the entrance wound path. The BCVA, IOFB size and shape, the wound, endophthalmitis, and silicone oil removal were compared among the three groups.

**Results:**

The IOFBs were 46 cases of magnetic and 27 cases of nonmagnetic, with a shape of thin flat in 19 cases, thick flat in 12 cases, long in seven cases, and irregular in 35 cases. Multiple damages were caused by the giant IOFBs, mainly involving the severe cornea, lens, and retina injuries. The postoperative BCVA increased compared with the preoperative BCVA (*z* = −6.06, *P* < 0.01). The rate of recovery from blindness was 40.85% (29/71). The thin flat IOFB and long IOFB resulted in a better postoperative BCVA than the other two IOFB shapes (all *P* < 0.05). The irregular IOFB had a poorer silicone oil removal rate than the other three IOFB shapes (all *P* < 0.05). The IOFB extraction followed the limbus path in 18 cases, pars plana path in 27 cases, and entrance wound path in 28 cases. The IOFB length and width in the pars plana path group were significantly lower than that in the limbus path group (all *P* < 0.05), the preoperative BCVA of the pars plana path group was superior to that of the limbus path group (*P* < 0.05), and the IOFB length, width, and entrance wound length in the pars plana path group were significantly lower than in the entrance wound path group (all *P* < 0.05). But the postoperative BCVA in the pars plana path group was not better than that in the other two groups (all *P* > 0.05). The postoperative BCVA of the entrance wound path group was significantly superior to that of the limbus path group (*z* = −2.01, *P* = 0.04), while there was no difference between the two groups in IOFB length, width, entrance wound length, or preoperative BCVA (all *P* > 0.05).

**Conclusion:**

The entrance wound path would benefit to minimize secondary injury in giant IOFB extraction procedure, compared with the limbus and pars plana path.

## Introduction

Ocular trauma is a major cause of blindness (1). Intraocular foreign body (IOFB) is a common type of open global injury with an occurrence rate of 28.60% (2). It was one of the chief causes of poor visual acuity prognosis in open globe injuries (3). IOFBs can cause mechanical, chemical, and biological injuries to the eye (4). The manifestations and prognosis of IOFB injuries vary depending on IOFB size, characteristics, and environment. Furthermore, the severity of mechanical eye damage by IOFB is related to its own size, weight, and kinetic energy (5), and the risk of poor visual acuity prognosis has been reported to increase 1.21-fold for every 1 mm of addition in IOFB length (6). Giant IOFBs cause both penetrating and blunt eye injuries including prolapse of intraocular contents and severe retinal and choroidal damage. Surgery for the extraction of giant IOFBs is one of the most difficult operations for eye injuries, as secondary injuries inevitably occur during the extraction procedure (7). A combined surgery with minimal incision pars plana vitrectomy (PPV) and giant IOFB extraction has been developed with the aim of decreasing secondary injury during IOFB extraction (8, 9). In this study, we analyzed the clinical manifestation and treatment of giant IOFB injuries using various extraction paths to discuss the best methods for decreasing secondary injuries during surgical extraction of giant IOFBs.

## Materials and Methods

### General Materials

We conducted a retrospective cohort study on a series of giant IOFB injuries. Seventy-three patients with giant IOFB injuries (73 eyes) were hospitalized for surgical therapy in the Fundus/Ocular Trauma Diseases Department at the First Affiliated Hospital of Zhengzhou University between January 2016 and December 2018. This study met the requirements of the Declaration of Helsinki and was approved by the Ethics Committee of the First Affiliated Hospital of Zhengzhou University, and the patients had provided informed consent.

The inclusion criteria were the following: First, the IOFB must be giant, defined as that with a length ≥10 mm, width ≥4 mm, (or) thickness ≥3 mm (10). Second, the patient's required first-aid therapy at our hospital after the eye injury, or the patients were transferred to our hospital within 5 days after debridement and suture surgery at other hospitals. Third, the patients had no other severe eye disease, except for IOFB eye injury. Fourth, none of the patients had any systemic diseases. The patients with any of the following manifestations were excluded from our observation group. First, the IOFB was incarcerated in the entrance wound so that part of it was exposed outside the eyeball. Second, the patients underwent evisceration during emergency debridement and suture surgery. Third, the patients had manifested most or all the retina prolapsed out of the injured eye in primary or secondary surgery. Fourth, the length of the IOFB entrance wound ≤ 3 mm. Fifth, the patients with concomitant diseases such as craniocerebral injury or other systemic organ injuries, systemic organic diseases, primary eye diseases, or cases lost to follow-up.

### Surgical Method and IOFB Extraction Path

The patients routinely underwent emergency debridement and suture procedures after the injury. The second phase of giant IOFB extraction combined with vitrectomy and global reconstruction was performed subsequently. This included the following surgical procedures according to the necessity of global reconstruction, such as giant IOFB extraction, vitrectomy, lensectomy, epiretinal membrane peeling, retinal photocoagulation/cryocoagulation, and intraocular silicone oil tamponade. All patients accepted silicone oil tamponade because of the severe ocular damage.

If proliferative vitreoretinopathy was observed during follow-up, the third surgery for epiretinal membrane peeling would be performed. The silicone oil removal surgery was performed when the globe was stable and the retina was restored for ≥3 months. All patients in this study were followed up for ≥6 months after the latest surgery.

**Figure 1:**
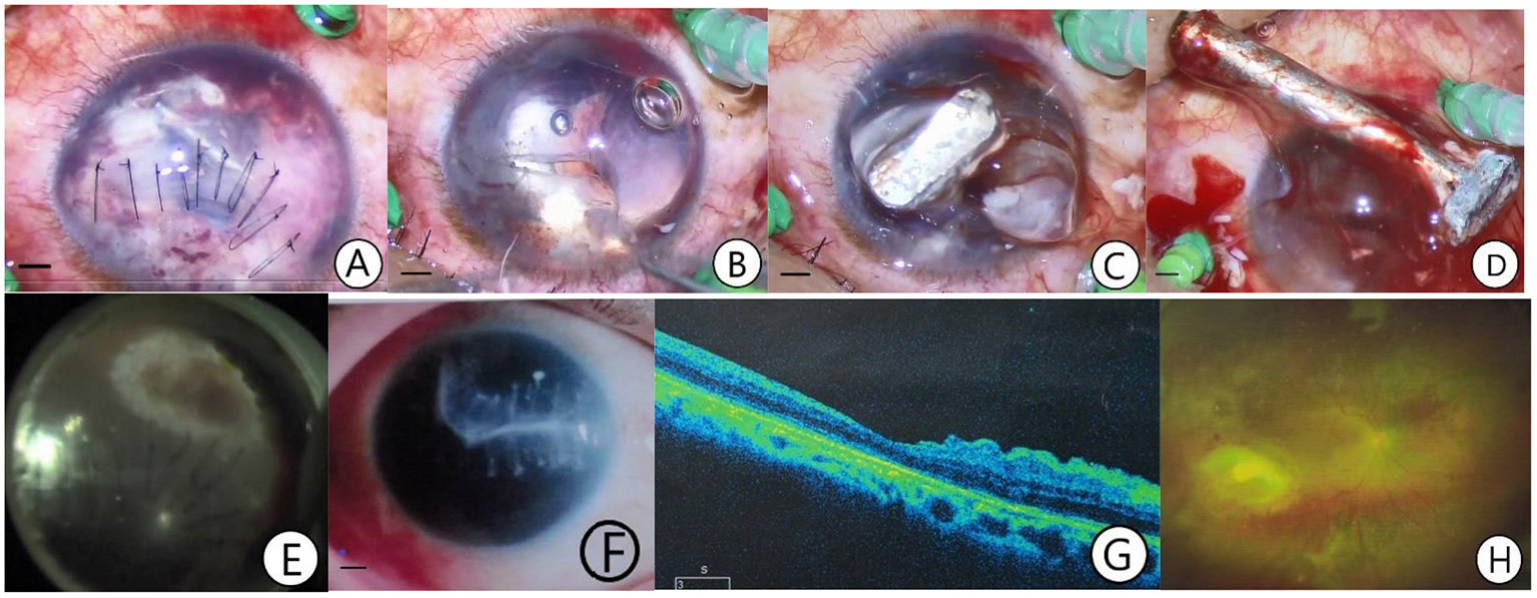
A case of giant IOFB extracted from the entrance wound path. The patient was a 41-year-old man admitted to the hospital 8 h after an eye injury caused by a nail. An emergency debridement and suture procedure were performed, and the combined second phase surgery of giant IOFB extraction from the entrance wound path, pars plana vitrectomy, retinectomy, retinal photocoagulation, and silicone oil tamponade was performed 24 h after the injury **(A–E)**. The silicone oil was removed in 4 months. The retina was recovered with a scar formation at the retinal wound caused by the IOFB. A postoperative BCVA of 0.1 was achieved **(F–H)**. **(A)** One end of the giant IOFB was exposed after the infiltration and the cortex of the ruptured lens was removed. **(B)** The exposed IOFB end was visible after the wound sutures were removed. **(C)** After intraocular viscoelastic solution injection and loosening the giant IOFB from the intraocular tissue, the head of the IOFB floated out of the wound. **(D)** The giant IOFB was extracted through the entrance wound. **(E)** Retinal photocoagulation was performed at the site of the retinal wound due to the IOFB. **(F)** Corneal scar formation at the IOFB entrance wound. **(G)** Optical coherence tomography macular image after silicone oil removal. **(H)** Fundus photograph after silicone oil removal showing that the retina had reattached stably with a scar formed at the retinal wound site (The length of the bar: 1 mm).

There were three paths for IOFB extraction in this study: the limbus path, the pars plana path, and the entrance wound path. In the limbus path procedure, a limbus tunnel incision was prepared after vitrectomy and lensectomy. The IOFB was then lifted into the anterior chamber or the pupil area using intraocular forceps, and another forceps was placed into the anterior chamber from the limbus tunnel incision to relay the IOFB and pull it out of the eyeball. A magnetic IOFB could also be relayed and extracted using a magnetic rod. Part of the patients underwent IOFB extraction using a pars plana incision path. After vitrectomy, the proliferating membrane around the IOFB was released. The pars plana incision for vitrectomy was enlarged, and the IOFB was extracted from this incision using forceps or a magnetic rod. As to the entrance wound path for IOFB extraction, vitrectomy and lensectomy were performed first; then, the IOFB was released from its surrounding proliferative membrane, and the wound suture of the stitched cornea (or the partial anterior sclera) was removed. The IOFB was subsequently lifted into the anterior chamber using the above method, except that the IOFB was extracted from the eye through the reopened entrance wound. After IOFB extraction, the wound was sutured. After that, the subsequent surgical procedures were finished. If the giant IOFB was so large that it impeded intraocular operation (Figure 1), the IOFB could be extracted before vitrectomy. Intraocular viscoelastics injection could be helpful for the IOFB extraction procedure under that condition.

### Observation Items and Groups

The best corrected visual acuity (BCVA), intraocular pressure (IOP), ocular tissue injuries, wound location and length, and intraoperative and postoperative complications were evaluated, together with the IOFB size, localization, shape, and magnetic properties. The interval time from injury to the second phase of IOFB extraction and ocular reconstruction, the IOFB extraction path, the rate of silicone oil removal, and globe recovery were also recorded for statistical analysis.

The patients in this study were divided into three groups according to the giant IOFB extraction path: the limbus path group, pars plana path group, and entrance wound path group. The IOFB size, shape, and magnetic properties as well as the preoperative and postoperative BCVA, length of the entrance wound, endophthalmitis, and rate of silicone oil removal were compared among the three groups.

In addition, to evaluate the IOFB shape distribution in the three IOFB extraction paths, the patients were also divided into four groups according to the giant IOFB shape: the thin flat IOFB group, the thick flat IOFB group, the long IOFB group, and the irregular IOFB group. IOFBs with a thickness < 1 mm and a width ≥5 mm were classified into the thin flat IOFB group. IOFBs with a thickness of 1–3 mm and a width ≥4 mm were classified into the thick flat IOFB group. IOFBs with a thickness ≥3 mm and a width > 3 mm were classified into irregular IOFB groups. Finally, IOFBs in which both the width and thickness were < 3 mm with a length ≥10 mm were classified into the long IOFB group. The preoperative and postoperative BCVAs and silicone oil removal of the four groups were compared.

### Statistical Analysis

The statistical analysis was completed using Statistical Package for Social Sciences software, version 21.0 (IBM, Chicago, IL). Quantitative data are provided in the form of [mean ± SD]. The BCVA, recorded in decimal form, was transformed into quantitative data by the logarithmic minimum angle of resolution (log MAR) for statistical analysis. Quantitative data meeting the normal distribution were analyzed using a *t*-test for comparison of paired samples, a *t*-test for group-designed two-sample mean comparison, or one-way ANOVA. Non-normally distributed quantitative data were tested using a nonparametric test (Kruskal-Wallis H test; Wilcoxon test). Qualitative data were tested using the chi-square test (χ^2^*-*test). Statistical significance was set at a *P* < 0.05.

## Results

### IOFB Characteristics and Eye Injuries

This study included 68 men and 5 women. Forty-three patients were injured in the right eye and 30 in the left eye. The average age of the patients was 34 ± 15 years, ranging between 1 and 63 years. In addition, 67.12% of all patients were aged between 21 and 50 years.

The giant IOFBs were magnetic and nonmagnetic in 46 and 27 cases, respectively. There were 19 cases of thin flat IOFBs, 12 cases of thick flat IOFBs, 7 cases of long IOFBs, and 35 cases of irregular IOFBs. The average IOFB length was 11.80 ± 4.85 mm, and the average IOFB width was 5.27 ± 1.85 mm. The average length of the entrance wound was 8.45 ± 3.95 mm (Table 1).

**Table 1:** Characteristics of giant intraocular foreign bodies (IOFBs) and injuries to the eye.

**Observation items**	**Classification**	**Cases (n)**	**%**
**MAGNETIC PROPERTIES OF GIANT IOFBs**			
Magnetic	Iron pieces	35	47.95
	Nails	11	15.07
Nonmagnetic	Glass	12	16.44
	Plastics	9	12.33
	Stone	3	4.11
	Copper pieces	2	2.74
	Bamboo	1	1.37
**SHAPE OF GIANT IOFBs**			
	Thin flat IOFBs	19	26.03
	Thick flat IOFBs	12	16.44
	Long IOFBs	7	9.59
	Irregular IOFBs	35	47.95
**IOFB LOCALIZATION**			
	Anterior segment	1	1.37
	Vitreous cavity	70	95.89
	Subretinal space	2	2.74
**INJURIES TO THE EYE**			
	Corneal wound	54	73.97
	Iris prolapse/incarceration	29	39.73
	Hyphemia	45	61.64
	Hypopyon/anterior chamber inflammation	27	36.99
	Broken of lens	54	73.97
	Dislocation/hemidislocation of lens	9	12.33
	Vitreous hemorrhage	69	94.52
	Vitreous abscess	15	20.55
	Retinal wound with subretinal hemorrhage	55	75.34
	Retinal detachment	46	63.01
	Retinal infection	25	34.25
	Retinal ischemia	7	9.59
	Endophthalmitis	27	36.99

Most cases in this study suffered multiple damages to the eye, including the cornea, iris, lens, vitreous body, retina, and choroid. Damage by giant IOFBs manifested as combined injuries of the anterior and posterior segments. The giant IOFBs caused corneal wounds in 54 patients (73.97%), mostly located in the vitreous cavity (95.89%, 70/73), among which 55 (75.34%, 55/73) cases had a direct wound on the retina. Those IOFBs hit the retina and were incarcerated in the inner wall of the globe, or fell into the vitreous after colliding with the retina. Most IOFBs caused multiple damages to the anterior and posterior segments of the eye, including the cornea, iris, lens, vitreous, and retina. Only two cases had no retinal damage in this study. The injuries to the eye are listed in Table 1.

### Surgeries and Outcomes

After emergency debridement and suture surgery, all patients underwent a combined surgery of IOFB extraction, vitrectomy, retinal photocoagulation (or cryocoagulation), and silicone oil tamponade. By the end of the follow-up period, 47 patients maintained a normal globe shape and had a stable recovery from the injury after the last silicone oil removal surgery. However, 25 patients did not undergo silicone oil removal because of a poor ocular condition, and one patient required ocular evisceration because of bullous keratopathy.

The cause for the failure to remove silicone oil from the globe was severe eye damage. Nine patients had large corneal wounds running through the center of the cornea combined with serious post-polar retinal injury, which resulted in poor postoperative visual acuity, with difficulties in postoperative fundus observation and postoperative proliferation management. Eight cases had a massive globe rupture due to the giant IOFBs, combined with prolapse of intraocular contents and destruction of intraocular structures. Five patients had severe corneal stroma opacity and edema, which delayed the second phase surgery for ocular reconstruction and created difficulty in globe reconstruction because of serious proliferative vitreoretinopathy. Two patients had serious endophthalmitis, intraocular empyema, and ocular tissue necrosis. One patient developed persistent ocular hypotension and *atrophia bulbi* because of anterior proliferative vitreoretinopathy and cyclitic membrane formation.

Other postoperative complications included postoperative proliferation and epiretinal membrane formation, retinal detachment, corneal endothelial decompensation, and secondary glaucoma. Twelve patients were complicated with postoperative epiretinal membrane, which was removed in a secondary surgery without recurrence. Four patients had complicated postoperative retinal detachment with proliferative epiretinal membrane, they had undergone subsequent surgery of retinal reattachment, epiretinal membrane peeling and silicone oil tamponade, and a stable retina reattachment was achieved after final silicone oil removal surgery. One patient with mild corneal endothelial decompensation after surgery had recovered after medical therapy and silicone oil removal. In addition, three cases with postoperative glaucoma had restored normal IOP after antiglaucoma drug treatment or silicone oil removal.

### Preoperative and Postoperative BCVA

The preoperative BCVA in this study showed no light perception (NLP) in three cases, from light perception (LP) to hand movement (HM) in 63 cases, from counting fingers (CF) to 0.04 in 5 cases, and from 0.05 to 0.25 in 2 cases. The average preoperative BCVA (logMAR) was 2.46 ± 0.33 (Table 2). The postoperative BCVA showed NLP in 3 cases, from LP to HM in 26 cases, from CF to 0.04 in 13 cases, from 0.05 to 0.25 in 29 cases, and > 0.3 in 2 cases. The average postoperative BCVA (logMAR) was 1.73 ± 0.79, and the rate of freedom from blindness was 40.85% (29/71; Table 2). Therefore, the BCVA significantly improved after surgical treatment (*z* = −6.06, *P* < 0.01; Table 2).

**Table 2:** Comparison of the preoperative and postoperative BCVAs for all and each giant IOFB extraction path.

**Groups**	**Cases (n)**	**Preoperative BCVA(n)**					**Postoperative BCVA (n)**					***Z* ^a^**	***P* ^a^**
		**NLP**	**LP **~**HM**	**CF **~**0.04**	**0.05**~**0.25**	**≥0.3**	**NLP**	**LP **~**HM**	**CF **~**0.04**	**0.05**~**0.25**	**≥0.3**		
Limbus path group	18	2	16	0	0	0	2	8	4	4	0	−2.53	0.01
Pars plana path group	27	0	20	5	2	0	1	10	2	7	2	−3.62	< 0.01
Entrance wound path group	28	0	28	0	0	0	0	8	8	12	0	−4.13	< 0.01
Total	73	3	63	5	2	0	3	26	13	29	2	−6.06	< 0.01

a *Comparison between the logMAR value of preoperative BCVA and postoperative BCVA, Wilcoxon signed-rank test; NLP, no light perception; LP, light perception; HM, hand movement; CF, counting finger*.

In addition, the preoperative and postoperative BCVAs compared in the three different IOFB extraction path groups showed that the postoperative BCVA was significantly better than the preoperative BCVA in each group (all *P* < 0.05; Table 2). In IOFB shape-based groups, the postoperative BCVA was also significantly better than the preoperative BCVA in each group (all *P* < 0.05; Table 3).

**Table 3:** Preoperative BCVA, postoperative BCVA, and rate of silicone oil removal in different IOFB shape groups.

**Groups**	**Cases (n)**	**BCVA (logMAR)**				**Silicone oil removal ^d^ (n)**	
		**Preoperative^a^**	**Postoperative^b^**	***Z* ^c^**	***P* ^c^**	**Yes**	**No**
Thin flat IOFB group	19	2.36 ± 0.44	1.10 ± 0.41	−3.83	<0.01	18	1
Thick flat IOFB group	12	2.55 ± 0.17	2.07 ± 0.62	−2.41	0.02	10	2
Long IOFB group	7	2.16 ± 0.57	1.21 ± 0.84	−2.21	0.03	6	1
Irregular IOFB group	35	2.56 ± 0.18	2.05 ± 0.75	−3.55	<0.01	13	22
*χ^2^* ^e^		8.89	20.96			22.29	
*P*		0.03	<0.01			<0.01	

a *Preoperative BCVA of the thin flat IOFB group compared with that of the thick flat IOFB group and the irregular IOFB group, respectively (z = −2.04,P = 0.04;z = −2.34,P = 0.02);*

b *postoperative BCVA of the thin flat IOFB group compared with that of the thick flat IOFB group and the irregular IOFB group, respectively (z = −3.88,P < 0.01;z = −3.76,P < 0.01). The postoperative BCVA of the long IOFB group compared with that of the thick flat IOFB group and the irregular IOFB group, respectively (z = −2.10,P = 0.04;z = −2.36,P = 0.02);*

c *Wilcoxon signed-rank test for the paired data of the preoperative BCVA and postoperative BCVA in each IOFB shape group;*

d *silicone oil removal of the irregular IOFB group compared with the long IOFB group, the thin flat IOFB group, and the thick flat IOFB group using Fisher's exact probability test (P < 0.01; P < 0.01; P = 0.03);*

e *comparison between the four IOFB shape groups, Kruskal-Wallis H-test for the logMAR value of preoperative BCVA and postoperative BCVA, chi-square test for the silicone oil removal*.

### Comparison of BCVA and Silicone Oil Removal According to IOFB Shape

The preoperative BCVA, postoperative BCVA, and rate of silicone oil removal were significantly different among the four IOFB shape-based groups (all *P* < 0.05). The preoperative and postoperative BCVAs of the thin flat IOFB group were better than those of the thick flat group and the irregular IOFB group, respectively (*P* < 0.05). The postoperative BCVA of the long IOFB group was better than that of the thick flat group and the irregular IOFB group, respectively (*P* < 0.05). In addition, the rate of silicone oil removal in the irregular IOFB group was lower than that in the other three IOFB shape groups (*P* < 0.05; Table 3).

### Comparison Among Extraction Paths

There were significant differences among the three extraction path groups in preoperative and postoperative BCVA, IOFB length and width, and the entrance wound length (all *P* < 0.05; Tables 4, 5). Whereas, there was no difference in terms of age, left or right side of the eye, interval time from injury to the second phase surgery, entrance wound location, endophthalmitis, magnetic properties, and silicone oil removal among extraction paths (all *P* > 0.05; Tables 4, 5).

**Table 4:** Best corrected visual acuity (BCVA), age, side of eye, interval time from injury to second phase surgery, entrance wound location, endophthalmitis, and silicone oil removal of giant IOFB according to extraction path.

**Groups**	**N**	**Preoperative BCVA^b^ (logMAR) (mean ±SD)**	**Postoperative BCVA^c^ (logMAR) (mean ±SD)**	**Age (years, [mean ±SD])**	**Side of the eye (n)**		**Interval time (day, [mean ±SD])**	**Entrance wound location (n)**			**Endophthalmitis (n)**		**Silicone oil removal (n)**	
					**Right**	**Center**		**I**	**II**	**III**	**Yes**	**No**	**Yes**	**No**
Limbus path group	18	2.60 ± 0.15	2.06 ± 0.78	31.22 ± 17.31	6	12	7.56 ± 4.53	10	6	2	4	14	10	8
Pars plana path group	27	2.31 ± 0.48	1.63 ± 0.88	29.89 ± 16.40	12	15	7.41 ± 4.22	16	7	4	9	18	17	10
Entrance wound path group	28	2.51 ± 0.14	1.61 ± 0.66	39.07 ± 10.22	12	16	7.29 ± 4.72	12	14	2	14	14	20	8
Total	73	2.46 ± 0.33	1.73 ± 0.79	33.74 ± 15.01	30	43	7.40 ± 4.43	38	27	8	27	46	47	26
*χ^2^* ^a^		7.03	4.33	5.07	0.61		0.03	3.75			3.87		1.24	
*P* ^a^		0.03	0.12	0.08	0.74		0.98	0.44			0.14		0.54	

a *Comparison between the three IOFB extraction path groups (Kruskal-Wallis H test);*

b *pairwise comparison between the three IOFB extraction path groups, the preoperative BCVA in the pars plana path group was better than that in the limbus path group (z = −2.46, P = 0.01), no statistical difference between the other paired groups (P > 0.05);*

c *pairwise comparison between the three IOFB extraction path groups, the postoperative BCVA in the entrance wound path group was better than that in the limbus path group (z = −2.01, P = 0.04), no statistical difference between the other paired groups (P > 0.05)*.

**Table 5:** Intraocular foreign bodies size, shape, magnetic properties, and entrance wound length according to IOFB extraction.

**Groups**	***N***	**FB length^b^ (mm, [mean ±SD])**	**FB width^c^ (mm, [mean ±SD])**	**Entrance wound length^d^ (mm, [mean ±SD])**	**FB shape^e^ (n)**				**FB properties (n)**	
					**Thin flat**	**Thick flat**	**Long**	**Irregular**	**Magnetic**	**Nonmagnetic**
The limbus path group	18	12.83 ± 4.20	6.00 ± 1.75	8.22 ± 3.62	4	4	0	10	10	8
The pars plana path group	27	9.65 ± 3.13	4.11 ± 1.55	7.22 ± 3.62	5	4	7	11	15	12
The entrance wound path group	28	13.21 ± 5.89	5.93 ± 1.65	9.79 ± 4.17	10	4	0	14	22	6
Total	73	11.80 ± 4.85	5.27 ± 1.85	8.45 ± 3.95	19	12	7	35	47	26
*χ^2^* ^a^		7.25	16.92	7.27	12.48	3.99				
*P* ^a^		0.03	< 0.01	0.03	0.04	0.14				

a *Comparison between the three extraction path groups, Kruskal-Wallis H test for quantitative data, chi-square test for qualitative data;*

b *pairwise comparison between the three IOFB extraction path groups, the IOFB length in the pars plana path group was shorter than that in the limbus path group and the entrance wound path group, respectively (z = −2.59, P = 0.01; z = −2.01, P = 0.04), no difference between the latter two groups (P > 0.05);*

c *pairwise comparison between the three IOFB extraction path groups, the IOFB width in the pars plana path group was shorter than that in the limbus path group and the entrance wound path group, respectively (z = −3.32, P < 0.01; z = −3.65, P < 0.01), no difference between the latter two groups (P > 0.05);*

d *pairwise comparison between the three IOFB extraction path groups, the entrance wound length in the pars plana path group was shorter than that in the entrance wound path group (z = −2.80, P < 0.01), no difference was found between the other paired groups (P > 0.05);*

e *Fisher's exact probability test. After eliminating long IOFB cases, no difference in shape distribution among the three IOFB extraction path groups (χ^2^ = 1.35, P = 0.85)*.

Comparing between the pars plana path and the limbus path, both IOFB length (*z* = −2.59, *P* = 0.01) and width (*z* = −3.32, *P* < 0.01) in the pars plana path group were significantly lower than that in the limbus path group (all *P* < 0.05), and the preoperative BCVA of the pars plana path group was significantly better than that of the limbus path group (z = −2.46, *P* = 0.01). But no difference was identified between the two groups (*P* > 0.05; Tables 4, 5).

Comparing between the pars plana path and the entrance wound path, IOFB length (*z* = −2.01, *P* = 0.04), IOFB width (*z* = −3.65, *P* < 0.01), and the entrance wound length (*z* = −2.80, *P* < 0.01) in the pars plana path group were significantly smaller than that in the entrance wound path group (*P* < 0.05). But no difference was identified between the two groups (*P* > 0.05; Tables 4, 5).

Comparing between the limbus path and the entrance wound path, no difference was identified between the two groups in IOFB length or width, entrance wound length, or preoperative BCVA (all *P* > 0.05). But Wilcoxon test showed that the postoperative BCVA of the entrance wound path group was significantly better than that of the limbus path group (*z* = −2.01; *P* = 0.04; Tables 4, 5).

As to the IOFB shape distribution comparison, all of the long IOFBs were extracted from the pars plana path in this study. After eliminating the long IOFBs, no difference in the distribution of the remaining three shape IOFBs on extraction paths was observed (χ^2^ = 1.35, *P* = 0.85; Table 4).

In the 38 cases where the end of the entrance wound was limited to area I (the wounds did not involve the global wall outside of the cornea), the entrance wound lengths in the limbus path group, pars plana path group, and the entrance wound path group were 5.60 ± 0.52 mm, 6.40 ± 1.99 mm, and 8.67 ± 2.61 mm, respectively, with significant difference (χ*2* = 8.25; *P* = 0.02) among them. The entrance wound in the entrance wound path group was longer than that in the limbus and pars plana path groups (*z* = –2.604; *P* = 0.009; *z* = –2.296, *P* = 0.022), but there was no difference between the latter two groups (*z* = –0.812; *P* = 0.417).

## Discussions

The intraocular foreign bodies are different in their mass, size, kinetic energy, chemical and magnetic properties, and microorganism contamination. The variety of IOFB injuries makes difference in prognosis. Sometimes, a small IOFB that damages the optic nerve or macula can cause severe visual loss, while a large IOFB may have a better vision prognosis if important structures are free from injury. However, less probability of damaging important structures occurred in small and slender IOFBs, while more probability occurred in large IOFBs. To avoid biased judgment in this study, all giant IOFBs were selected strictly to meet the inclusion criteria size, and the IOFB incarcerated in the entrance wound was also excluded. The characteristics of this case series were a large proportion of huge cornea wound, lens broken, and retinal wound, which made the data comparable for IOFB extraction path analysis.

Similar to the previous reports (11–13), the giant IOFBs caused serious ocular structural damage in this study. Giant IOFB injuries mostly occurred in young and middle-aged men in this series, with 67.12% of them aged 21–50 years. Only 64.38% of the cases in this study underwent silicone oil removal surgery and maintained a stable ocular shape with partial restoration of visual acuity. The patients who recovered from blindness (final BCVA ≥0.05) accounted for 40.85% of all patients. Despite severe damage to the eyes, visual acuity improved in most cases after surgery. Therefore, improving management options for IOFB extraction may improve the prognosis.

At present, the treatment for IOFB injury includes single IOFB extraction surgery, or a combined IOFB extraction surgery, PPV, and other procedures for ocular reconstruction. IOFB extraction paths include the posterior scleral path through the IOFB location site, the pars plana incision path, the limbus tunnel incision path, and the IOFB entrance wound path (13–15).

Reducing secondary damage during giant IOFB extraction is important for improving visual prognosis. There were two main ways to reduce the secondary injury during IOFB extraction: one was to reduce the size of the incision for extracting the IOFB, and the other one was choosing the best incision site. Since the incision length was related to IOFB size, it was usually slightly wider than the IOFB width, leaving little room for intervention in this area. Therefore, the incision site selection was chosen to decrease the injury. A suitable incision site would allow smoother IOFB extraction with milder injury (15). The incision selection should consider the IOFB location, entrance wound location and size, the transparency of the cornea, and the condition of the lens (4). Previously, expanding the pars plana incision of PPV would be ideal for cases with an intact lens, while limbus incision would be preferred in cases with an injured lens (16).

The pars plana path extraction incision could avoid cornea injury and iris prolapse (17). But excessively large incision for a large IOFB increased the risk of retinal prolapse (18) and caused sudden and severely low IOP, which could induce corneal depression and deformation. An over-enlarged incision might also increase the risk of subchoroidal hemorrhage, ora serrata dialysis, retinal detachment, ciliary body injury, and postoperative low IOP (19). The limbus tunnel path provided a wide and direct view of the operation, avoiding injuries to the ciliary body or the ora serrata (14, 20). However, the lens must be removed for this extraction, and a large limbus incision could worsen corneal and anterior chamber angle injuries. Secondary glaucoma is more likely to occur in patients with anterior chamber angle trauma. Furthermore, limited by the anterior chamber depth, irregular IOFBs would abrade the endothelial cells of the cornea. The entrance wound path had more room for IOFB extraction, hence, no need of making a new incision (21). Thinking about the giant IOFB size, the secondary injury of an incision should not be ignored. However, the sutured entrance wound should be reopened and re-sutured, which spent more operational time. Occasionally, corneal wound leakage might occur in cases of corneal wound lysis.

When comparing the advantages of the three IOFB extraction paths in giant IOFB injury, still other prognostic factors should be considered. The wound length was consistent with the size of the IOFB and was associated with visual prognosis (2, 11). A poorer preoperative BCVA in giant IOFBs injuries would result in a poorer postoperative BCVA, and the larger the IOFB width/wound length, the poorer the visual acuity outcome (10). Damage to the eyes varied because of the different IOFB shapes in this series. The eyes injured by thin flat or long IOFBs have a better visual prognosis, but an irregular IOFB injury would result in a poor prognosis both in terms of visual acuity and global shape recovery.

In this series, the IOFB size and shape and the preoperative BCVA in the pars plana path group indicated a better prognosis. However, the postoperative BCVA and the rate of silicone oil removal in this group had no advantage over the limbus path group or the entrance wound path groups. The limbus path and the entrance wound path would be better choices for the extraction of giant IOFBs compared with the pars plana path.

When making a comparison between the limbus path and entrance wound path groups, no difference was found in the preoperative BCVA, IOFB size, IOFB shape, or the wound length. In the 38 cases in which the end of the wound was limited to the cornea, the IOFB length of the entrance wound path was longer than that of the limbus path group. However, the postoperative BCVA of the entrance wound path group was better than that of the limbus path group. Hence, an entrance wound path IOFB extraction would be more advantageous for a better prognosis.

The IOFB was a flying object before it entered the eye. It kept its long axis along its flight path, which reduced frictional resistance. As a result, the long axis of the IOFB was more likely perpendicular to the wound plane. An IOFB extraction from the entrance wound was coincident with this condition, avoiding new damage of another incision. Furthermore, most of the entrance wounds were in the anterior segment of the eye (zones I and II) in this case series, which facilitates this procedure. In particular, for very large IOFBs, the entrance wound path is the only choice. Two cases of very large IOFBs in this study had an unexpectedly good visual acuity by using the entrance wound path IOFB extraction, considering their severe eye damage.

Extraction of IOFB from the entrance wound path needs an in-time secondary phase surgery; otherwise, it would be difficult to reopen the sutured wound. The in-time surgery for IOFB extraction and ocular reconstruction could be beneficial for the success of retinal reattachment (16), as it would interrupt the continuous mechanical and chemical injury of IOFBs, and resolve the hemorrhage, retinal detachment, proliferation membrane, and endophthalmitis in the eye (22, 23). This might be another cause for a better prognosis in the entrance path group.

## Conclusion

In summary, giant IOFB injury can cause severe damage to the eye and result in a poor prognosis. However, postoperative BCVA could be improved after intensive treatment. IOFB extraction should obey the rule of minimum damage, considering the IOFB size, shape, and properties. IOFBs with a large width are not suitable for pars plana path extraction, whereas the entrance wound path would be more advantageous for giant IOFB extraction. In this study, we encountered several problems. The giant IOFBs are various in size and shape, which made the comparison difficult. Though we defined the giant IOFB size, there are still some inconformities in ocular damage caused by each IOFB. The lack of enough cases for analysis in this study would make us lose valuable information for judgment. Hence, more studies and discussions should be conducted on the surgical design and postoperative management to decrease secondary injury in patients with giant IOFB injuries.

## Data Availability Statement

The original contributions presented in the study are included in the article/supplementary material, further inquiries can be directed to the corresponding author.

## Ethics Statement

The studies involving human participants were reviewed and approved by the Ethics Committee of the First Affiliated Hospital of Zhengzhou University. Written informed consent to participate in this study was provided by the participants' legal guardian/next of kin.

## Author Contributions

JM had finished the statistical analysis and manuscript. All authors had taken part in the clinical procedure and data collection and agree to be accountable for the content of the study.

## Conflict of Interest

The authors declare that the research was conducted in the absence of any commercial or financial relationships that could be construed as a potential conflict of interest.

## Publisher's Note

All claims expressed in this article are solely those of the authors and do not necessarily represent those of their affiliated organizations, or those of the publisher, the editors and the reviewers. Any product that may be evaluated in this article, or claim that may be made by its manufacturer, is not guaranteed or endorsed by the publisher.

## Abbreviations

IOFB: intraocular foreign body
BCVA: best corrected visual acuity
IOP: intraocular pressure
NLP: no light perception
LP: light perception
HM: hand movement
CF: counting finger
OCT: optical coherence tomography.
